# Diabetes em Dia Program: acceptability and feasibility of the proposal

**DOI:** 10.15649/cuidarte.4268

**Published:** 2025-08-15

**Authors:** Maria Meimei Brevidelli, Edvane Birelo Lopes De Domenico, Gilcelli Vascom Girotto Rodrigues, Veronica Paula Torel de Moura

**Affiliations:** 1 Instituto de Ciências de Saúde, Universidade Paulista - UNIP São Paulo (SP), Brazil. E-mail: maria.brevidelli@docente.unip.br UNIP São Paulo (SP) Brazil maria.brevidelli@docente.unip.br; 2 Departamento de Enfermagem Clínica e Cirúrgica da Escola Paulista de Enfermagem, Universidade Federal de São Paulo (EPE-UNIFESP), São Paulo (SP), Brazil. E-mail: domenico.edvane@unifesp.br EPE-UNIFESP São Paulo (SP) Brazil domenico.edvane@unifesp.br; 3 Programa de Pós-Graduação no Ensino da Saúde, Centro de Desenvolvimento do Ensino Superior em Saúde, Universidade Federal de São Paulo (CEDESS-UNIFESP), São Paulo (SP), Brazil. E-mail: gilcellivgr1978@gmail.com CEDESS-UNIFESP São Paulo (SP) Brazil gilcellivgr1978@gmail.com; 4 Programa de Pós-Graduação da Escola Paulista de Enfermagem, Universidade Federal de São Paulo (EPE-UNIFESP), São Paulo (SP), Brazil. E-mail: veronica.moura@bp.org.br EPE-UNIFESP São Paulo (SP) Brazil veronica.moura@bp.org.br

**Keywords:** Diabetes Mellitus, Self-Management, Health Education, Supplementary Health, Feasibility Studies, Diabetes Mellitus, Autogestión, Educación para la Salud, Salud Complementaria, Estudios de Factibilidad, Diabetes Mellitus, Autogestão, Educação para Saúde, Saúde Suplementar, Estudos de Viabilidade

## Abstract

**Introduction:**

The contemporary approach to diabetes care emphasizes education for self-management.

**Objective:**

To evaluate the acceptability and feasibility of a proposed educational program for type 2 diabetes self-management (Diabetes em Dia Program), tailored for the users of an outpatient service at a private hospital in São Paulo, Brazil.

**Materials and Methods:**

This was an evaluation study of the preliminary proposal for a complex intervention conducted by health professionals from the hospital's Diabetes Study Group. After being introduced to the proposal, professionals were invited to assess it using a semantic differential scale based on criteria of acceptability and feasibility. Evaluation was based on agreement percentages, and consensus was achieved when agreement reached ≥ 89%.

**Results:**

There was consensus that the program is suitable for managing type 2 diabetes; it is understandable, acceptable, and effective. It was also considered to pose no risk to patients or health professionals and require minimal additional material resources and ongoing supervision. However, there was no consensus regarding the program's general assessment, the effort and time required for its application, its ease/difficulty of application, the need for additional human resources, and its cost-effectiveness.

**Discussion:**

Professionals recognized the program's importance, suitability, value, and positive effects. However, they acknowledged that effective implementation would require significant changes in work processes.

**Conclusion:**

Although the program was favorably assessed across many acceptability criteria, barriers to its implementation were identified.

## Resumen

**Introducción:**

El enfoque contemporáneo del cuidado de la diabetes guía la educación para el automanejo.

**Objetivo:**

Evaluar la aceptabilidad y viabilidad de la propuesta de un programa educativo para el automanejo de la diabetes tipo 2 (Programa Diabetes em Día), adaptado a los usuarios de un servicio ambulatorio de un hospital privado de la ciudad de São Paulo, Brasil.

**Materiales y métodos:**

Estudio de evaluación de la propuesta preliminar de una intervención compleja, realizada por profesionales de la salud miembros del Grupo de Estudio de la Diabetes del hospital. Tras conocer la propuesta, se invitó a los profesionales a evaluarla mediante una escala diferencial semántica con criterios de aceptabilidad y viabilidad. Se consideraron los porcentajes de acuerdo para la evaluación, y se alcanzó el consenso cuando el acuerdo fue ≥ 89%.

**Resultados:**

Hubo consenso en que el programa era adecuado para el manejo de la diabetes tipo 2; comprensible, aceptable y eficaz; no presentaba riesgos para la persona ni para el profesional de la salud; y requería pocos recursos materiales adicionales y supervisión constante. No hubo consenso sobre la evaluación general del programa; el esfuerzo y el tiempo requeridos para su implementación; la facilidad/dificultad de implementación; la necesidad de recursos humanos adicionales; y la rentabilidad de la propuesta.

**Discusión:**

Los profesionales demostraron ser conscientes de la importancia e idoneidad del programa, reconociendo su valor y sus efectos positivos. Sin embargo, reconocieron que su implementación efectiva requeriría cambios significativos en los procesos de trabajo.

**Conclusiones:**

Si bien el programa recibió una evaluación favorable en muchos criterios de aceptabilidad, presenta barreras para su implementación.

## Resumo

**Introdução:**

A abordagem contemporânea do cuidado em diabetes orienta a educação para o autogerenciamento.

**Objetivo:**

Avaliar a aceitabilidade e viabilidade da proposta de um programa educativo para autogerenciamento em diabetes tipo 2 (Programa Diabetes em Dia), personalizado para os usuários de um serviço ambulatorial, de um hospital privado na cidade de São Paulo, Brasil.

**Materiais e métodos:**

Estudo de avaliação da proposta preliminar de uma intervenção complexa por profissionais de saúde integrantes do Grupo de Estudos em Diabetes do hospital. Após conhecerem a proposta, os profissionais foram convidados a julgá-la, utilizando uma escala de diferencial semântico com critérios de aceitabilidade e viabilidade. Para avaliação foram consideradas as percentagens de concordância e, a obtenção de consenso quando a concordância foi ≥ 89%.

**Resultados:**

Houve consenso sobre o programa ser adequado para lidar com diabetes tipo 2; ser compreensível, aceitável e efetivo; não oferecer riscos a pessoa e ao profissional de saúde; exigir poucos recursos materiais adicionais e supervisão constantes. Não houve consenso quanto à apreciação geral do programa; esforço e tempo exigidos para aplicação; facilidade/dificuldade de ser aplicado; exigência de recursos humanos adicionais e sobre a proposta ser custo-efetiva.

**Discussão:**

Os profissionais demonstraram estar conscientes da importância e da adequação do programa, reconhecendo o valor e os efeitos positivos deste. Entretanto, perceberam que a implementação efetiva do programa exigiria mudanças significativas nos processos de trabalho.

**Conclusões:**

Apesar de o programa ter sido apreciado favoravelmente em muitos critérios de aceitabilidade, possui barreiras para ser implementado.

## Introduction

The contemporary approach to diabetes care emphasizes self-management, which is understood as the ability to make decisions and take action to enhance positive health outcomes. This approach involves developing skills to understand and manage the chronic condition, which includes empowering individuals to overcome barriers to self-care practices^1^,^2^. 

Diabetes Self-Management Education (DSME) involves developing a collaborative relationship between the person with diabetes and the health educator, aimed at enabling the individual to manage the daily demands of care. Scientific evidence supports the effectiveness of structured interventions that promote self-management in diabetes, resulting in glycemic control, improved quality of life, and coping well with the disease, especially among people with type 2 diabetes^3^.

The positive outcomes of DSME are attributed to the combination of multiple cognitive and behavioral approaches that encourage individuals with diabetes to participate and take responsibility for their own care. Thus, educational strategies have shifted away from being overly concerned with content aimed at informing and promoting compliance behaviors, toward didactic-pedagogical strategies that develop the technical, emotional, and psychosocial skills necessary to meet the complex care demands of diabetes illness^4^.

In this regard, strategies that include setting goals/objectives, developing action plans, collaborating on problem-solving, offering emotional support, and utilizing a support network (family, friends) have been considered effective^5^. This combination fosters empowerment in disease control, that is, personal self-efficacy, by building individuals' confidence in engaging in desirable behaviors^4^. 

In high-income countries, DSME is an essential component of diabetes care, whose cost-effectiveness surpasses other current practices and adds value to interdisciplinary care^5^-^7^. For this reason, it has become the educational standard of excellence in diabetes care in the United States, Canada, and the United Kingdom^8^-^11^. 

The X-PERT structured education program for individuals with type 2 diabetes, whether newly diagnosed or with established disease, has demonstrated positive outcomes in glycemic control, cardiovascular health, and the empowerment of its 3,376 participants over a one-year period^12^. A recent systematic review aimed at determining the clinical impact of DSME on people with diabetes living in low- and middle-income countries revealed an association between this approach and a reduction in glycemic control, particularly in Sub-Saharan Africa^13^.

In Brazil, to date, no proposals for a structured educational approach focusing on diabetes self-management and utilizing behavior change strategies have been identified in the scientific literature. To address this gap and considering the potential benefits of DSME, it was deemed relevant to outline an educational program to promote self-management among individuals with type 2 diabetes, known as the Diabetes em Dia Program (Dia-D Program). The proposal was based on scientific evidence and behavioral change models, and was personalized for users of a supplementary health service^14^. 

Data from the Panorama Supplementary Health bulletin for the third quarter of 2023 indicate that 50.9 million people were users of the supplementary health system for medical and hospital care, accounting for BRL 1.6 billion in expenses related to procedures and hospitalizations^15^. Recent data from this population indicated that 6.9% of people with health plans reported a medical diagnosis of diabetes, and this prevalence increased with age, reaching 19% among those aged 65 years old^16^. While these users tend to have better health indicators, such as higher consumption of healthy foods and greater access to preventive screenings, regardless of education level^17^, they also experience vulnerabilities that must be considered in the presence of chronic diseases.

A recent study conducted with this population identified inconsistent adherence to diabetes self-care among a group composed predominantly of older adults, who have various vulnerabilities such as overweight/obesity, chronic complications associated with type 2 diabetes, and a negative attitude towards self-care. Moreover, the correct use of medication was the behavior with higher adherence, whereas lifestyle changes, such as physical exercise, had lower adherence^18^. These results revealed that the complexity of care inherent to diabetes, even among individuals with higher education and income^17^, underscores the need for a structured educational process tailored to this population. 

This research was motivated by the need for greater investment in health education programs based on the principles of DSME for the Brazilian population, particularly those who rely on supplementary health services. From this perspective, the study aimed to evaluate the acceptability and feasibility of a proposed educational program for self-management of type 2 diabetes (Diabetes em Dia Program), tailored for the users of an outpatient service at a private hospital in São Paulo, Brazil.

## Materials and Methods

This was an evaluation study of the preliminary proposal for a complex intervention conducted by health professionals from the hospital's Diabetes Study Group. The intervention project was registered on the Brazilian Registry of Clinical Trials platform (https://ensaiosclinicos.gov.br/rg/RBR-8nx9n4d). The term "complex" refers to interventions composed of multiple components or mechanisms of change that depend on interactions with the environment or context in which they are implemented^19^.

According to the latest guidelines from the United Kingdom Medical Research Council^19^,^20^, the development of a complex intervention should consider as one of its essential elements the participation of people who will be affected by the intervention (stakeholders), since such involvement contributes to increasing its implementability in the context of practice^21^. 

The integration of different stakeholders, including non-research participants, contributes to adding a realistic and non-academic perspective to the process, thereby increasing the chances of the intervention's effectiveness. Therefore, it is a collaborative process between researchers and participants, which, due to the complexity of the intervention, depends on this collaborative interaction to enhance the likelihood of success during its implementation^22^. 

In participatory research, the level of collaboration can vary considerably, ranging from simply informing participants to fully delegating decision-making about the process^22^. In this study, collaboration was based on obtaining feedback on the preliminary proposal of the educational intervention. The description of the intervention development process followed the recommendations of the Guidance for Reporting Intervention Development Studies in Health Research (GUIDED)^21^ and the Template for Intervention Description and Replication (TIDieR) checklist^23^. 

For the collection of empirical data, the guidelines and regulatory standards for research involving human subjects, as approved by the National Health Council in Resolution 466/12, were followed. The study was submitted to the Research Ethics Committee of the hospital where the outpatient service is located (CAAE 56916722.0.0000.5455) and was approved in July 2022 (Opinion: 5,543,375).

**Characteristics of the preliminary intervention proposal**

The preliminary intervention proposal was developed by combining the Seven Self-Care Behaviors™ from the Association of Diabetes Care & Education Specialists (ADCES7)^24^, with the Behaviour Change Wheel (BCW)^25^.

The intervention was grounded in the premise that effective self-management of type 2 diabetes requires adherence to at least three fundamental self-care behaviors: healthy eating, being active, and taking medication. The selection of these behaviors took into account their direct relationship with clinical indicators of disease control, specifically the reduction of HbA1C, body weight, and abdominal circumference^26^. Represented with concentric rings in the circular graphic representation of the ADCES7 Self-Care Behaviors™, these three behaviors underpin the essential care that a person with diabetes should regularly adopt and, therefore, should be considered basic in any care plan^24^. Additionally, by working with behavior change techniques, promoting personal self-efficacy, and adopting a person-centered approach, the Dia-D Program implicitly incorporates the remaining ADCES7 Self-Care Behaviours: healthy coping, monitoring, problem solving, and reducing risks. 

Table 1 presents the definitions for each target behavior, based on the Clinical Standards of Diabetes Care from the American Diabetes Association and the ADCES7^24^,^26^. 

**Table 1:** Description of the Target Behaviors for the Educational Intervention, 2022

Target behaviors	Description
Healthy eating	Balanced carbohydrate consumption through portion control, encouragement of low-glycemic-index carbohydrate intake, and replacement of saturated fats with monounsaturated fats.
Being active	Engage in 150 minutes per week of regular and progressive activities such as brisk walking, or increase daily step count (up to 10,000 steps/day).
Taking medications	Strict adherence to the prescribed medication regimen (correct medication, route, dose, and timing).

The development of the intervention was informed by scientific evidence on the characteristics and factors that support its effectiveness. These include the use of digital platforms to engage participants (e.g., internet-based programs, telemedicine, mobile apps, remote monitoring), the incorporation of psychosocial aspects and the influence of beliefs into the intervention curriculum, 10 hours of interaction with participants, a combination of individual interventions that consider personal needs and preferences, a person- and family-centered educational approach, and a focus on behavior change techniques^4^,^11^,^26^-^27^. 

The Behaviour Change Wheel (BCW) was used to select behavior change techniques through the analysis of the elements of the behavioral system, known as COM-B (Capability, Opportunity, Motivation-Behaviour). This framework made it possible to identify which internal conditions, within the individual and their physical and social environment, must be present for a particular behavior to occur, as well as the aspects of the motivational system that need to be activated^14^. 

A recent systematic review on the barriers and facilitators to the successful management of type 2 diabetes, based on studies conducted in Latin America and the Caribbean, including Brazil, served as a foundation for identifying the COM-B behavioral system^28^. Further details on the development process of the preliminary Dia-D Program proposal can be found in another study^14^. 

The proposal was tailored for adults with type 2 diabetes, whether newly diagnosed or with established disease, who are users of a Kidney and Diabetes Center that is part of a private hospital recognized for providing high-complexity care in central São Paulo, Brazil. To participate in the program, users need to have access to digital communication platforms and demonstrate digital proficiency. Pregnant women and individuals with cognitive or functional limitations that hinder lifestyle changes (e.g., those with severe depression, renal failure, retinopathy, and diabetic neuropathy) were not eligible to participate. 

The selection of a private institution was made both due to the scarcity of research involving participants from this healthcare setting and the presence of an endocrinology department within the institution, capable of ensuring the operability of the research stages. It is worth noting that the study originated from a shared interest between the researchers and the institution's management.

This center aims to provide early diagnosis, prompt treatment, and multidisciplinary care in a single location, where consultations, exams, and outpatient procedures for diabetes treatment can be conducted. It is staffed by a team of nephrologists, urologists, endocrinologists, nurses, and nutritionists.

Generally, users of this service seek medical care from a physician affiliated with their health insurance plan provider, and this professional determines the treatment protocols and guidance, which may be based on clinical guidelines or their own judgment as a specialist. Even when a health educator, nurse, or nutritionist is involved, their engagement is related to issues presented by the users, such as frequent hypoglycemia, weight gain, or difficulty adhering to the prescribed diet. 

The implementation proposal includes formal training for healthcare professionals who will deliver the intervention, covering the following content: clinical guidelines for diabetes care, principles of person- and family-centered care and self-management in diabetes, and how to conduct Dia-D Program educational sessions. Table 2 presents the components of the Dia-D Program

**Table 2:** Components of the Dia-D Program, 2022

Components	Description and Purpose
Digital platforms	Interaction with participants via digital communication platforms to facilitate access to the program.
Educational sessions	Six consecutive sessions involving interaction between participants and the health team (total=10h) were designed to shape knowledge and build skills, promote self-efficacy in diabetes, create an environment conducive to achieving target behaviors, and provide participant monitoring.
Supportive educational material	Educational videos on healthy eating, physical activity programs, and the correct use of medications are used to present and reinforce learning. A diary for recording and monitoring adopted behaviors (Diabetes em Dia Diary) and a workbook aimed at increasing the ability to solve everyday problems and enhance personal self-efficacy (Diabetes Day by Day: Discovering Solutions).
Health team service script	Material to guide the health team in delivering the intervention modules, aiming to ensure fidelity to the intervention.

**Participants**

To evaluate and improve the preliminary proposal, the sample consisted of health professionals who provide routine care and conduct educational activities for people with diabetes within the health service. Therefore, members of the multidisciplinary team that forms the Diabetes Study Group at the hospital, where the outpatient service is located, were chosen. This group included nurses, nutritionists, and pharmacists. 

The recruitment of the professionals was conducted internally at the hospital through an announcement made during a study group meeting. The eligibility criteria were as follows: (i) to be active in the hospital's study group; (ii) to have the availability to attend a single virtual meeting to learn about the preliminary proposal, with the date and time communicated in advance. The only exclusion criterion was failure to complete all required steps, including full participation in the meeting and submission of all the forms. 

**Data collection and instruments**

The professionals were invited to a 60-minute virtual meeting during which the principal investigator presented the details of the preliminary proposal: theoretical-conceptual foundations on education for diabetes self-management, the theoretical models underpinning the preliminary proposal, the program components tailored to the outpatient service, the selected behavior change techniques, and the content of the interactive sessions. A single meeting was enough, as the participants expressed satisfaction with the presented proposal and understood its content before the session concluded. 

After the meeting, participants received a link to an online form hosted on the Google Forms® platform, which included the informed consent form and a questionnaire to provide feedback on the proposal based on predetermined criteria of acceptability and feasibility formulated from items described in the literature for these constructs^29^-^31^. Supplementary materials related to data collection are available in the Mendeley repository^32^. The questionnaire collected the following data:

**- Sociodemographic and professional information:** age, gender, professional category, academic qualifications, participation as a member of an organization specializing in diabetes care/education, professional experience, time spent in diabetes care/education, and professional activity at the study hospital.

**- Acceptability:** Assessed using a semantic differential scale (1 to 10 points) to evaluate program attributes described by Sekhon et al.^29^-^30^. The attributes included: overall appreciation, suitability for dealing with type 2 diabetes, professional effort to implement the intervention, ease/difficulty of application as designed, potential harm to individuals with type 2 diabetes, potential risk to health professionals, effectiveness, understanding of the mechanism of action, and general acceptability.

**- Feasibility:** Assessed using a semantic differential scale (1 to 10 points) to evaluate program attributes described by van der Krieke et al.^31^. The attributes included time required for application, need for ongoing support and supervision, additional human resources required, additional material resources required, and cost-effectiveness.

**Data analysis**

Descriptive statistics were used to present the sociodemographic and professional characteristics of the participants. To assess the level of agreement among participants regarding the program's acceptability and feasibility, the criterion defined in an article on consensus thresholds in the Delphi technique was used. According to this criterion, the specific number of participants, which can vary from two to ten, indicates the percentage of agreement that must be achieved to ensure consensus with a statistical confidence level >95%. Following this criterion, in the present study, consensus was considered when the agreement was ≥ 89%^33^. Additionally, the percentages of agreement were calculated for three levels of the semantic differential scales: weak agreement (scores from 1 to 4), moderate agreement (scores from 5 to 7), and strong agreement (scores from 8 to 10). The analysis also included measures of central tendency and dispersion, such as the median, interquartile range, and minimum and maximum values. 

## Results

Nine health professionals agreed to participate in the study, distributed across three professional categories: nurses, nutritionists, and pharmacists, each group constituting one-third of the sample. One professional who attended the meeting about the program declined to complete the questionnaire. The sociodemographic and professional characteristics of the participants are described in Table 3.

**Table 3:** Sociodemographic and Professional Data of Participants, São Paulo (SP), 2022

Sociodemographic and professional data	Frequency N= 09 %(n)
Gender	
Female	88.89 (8)
Male	11.11 (1)
Age (years old)	
Mean (SD^§^)	38.44 ± 9.33
Median (IQR*)’	39.00 (26.00)
Min - Max	27.00 – 53.00
Professional Category	
Nurse	33.33 (3)
Pharmacist	33.33 (3)
Nutritionist	33.33 (3)
Academic degree	
Specialist	77.80 (7)
Diabetes Expert and Educator	22.20 (2)
Affiliation with the Association of Diabetes Specialists	
Brazilian Diabetes Society (SBD)/ Juvenile Diabetes Association (ADJ)	11.11 (1)
None	88.89 (8)
Length of Professional Practice	
1 - 5 years	11.11 (1)
6 - 10 years	44.44 (4)
11 - 15 years	22.22 (2)
16 - 20 years	11.11 (1)
> 20 years	11.11 (1)
Time Practicing as a Diabetes Specialist	
1 - 5 years	55.56 (5)
6 - 10 years	44.44 (4)

§SD = Standard Deviation; *IQR = Interquartile Range

There was a significant predominance of female professionals (88.89%), with a mean age of 38.44 years (SD=9.33). The majority declared holding a specialist degree (77.80%), while the remainder identified themselves as diabetes specialists and diabetes educators (22.20%). Regarding the length of professional practice, 44.44% had between 6 and 10 years of experience, and the same frequency of professionals was observed when combining the categories of those with 11 years of experience or more. As for the time spent as diabetes specialists, all participants had between 1 and 10 years of experience, with just over half (55.56%) reporting between 1 and 5 years. 

Regarding the acceptability evaluation of the Dia-D Program (Table 4), there was consensus, that is, strong agreement from at least 88.9% of participants, on the following aspects: "extremely suitable" for managing type 2 diabetes, "completely effective," with a "fully understandable" mechanism of action, and "completely acceptable" overall. Furthermore, all participants considered it "not at all likely" that the program would cause harm to individuals with diabetes or pose any risk to health professionals. It is also notable that, although consensus was not reached for the items related to overall appreciation, required effort, and difficulty of application, the majority "strongly liked" it (77.8%), considered its implementation would involved "huge effort" (77.78%), yet found it "easy to apply" (66.67%). 

In the feasibility evaluation of the proposal (Table 4), there was consensus on the "need for ongoing support and supervision" and the absence of a need for "additional material resources." Despite the absence of consensus, a significant part of the participants considered that the program would require "an extremely long time to implement" (55.56%), "a moderate increase in human resources" (44.45%), and that the proposal is "completely cost-effective" (77.78%).

**Table 4:** Acceptability and feasibility scores of the Dia-D Program, São Paulo (SP), 2022

	Level of Agreement			Median (IQR)*	Min- Max^§^
	Weak (1-4) % (n)	Moderate (5-7) % (n)	Strong (8-10) % (n)		
Acceptability					
Overall appreciation (strongly disliked/strongly liked)	-	22.22 (2)	77.78 (7)	10.00 [7.50 ; 10.00]	7.00 -10.00
Suitability for managing type 2 diabetes (extremely unsuitable/extremely suitable)	-	11.11 (1)	88.89 (8)	10.00 [8.50 ;10.00]	7.00 -10.00
Effort required for application (no effort at all/huge effort)	11.11 (1)	11.11 (1)	77.78 (7)	8.00 [6.50 ; 9.50]	3.00 -10.00
Difficulty/Ease of application (hardly applicable/easily applicable)	33.33 (3)	-	66.67 (6)	8.00 [3.50 ; 9.00]	2.00 -9.00
Possibility of harm to the person with type 2 diabetes (not at all likely/totally likely)	100.00 (9)	-	-	1.00 [1.00 ; 1.50]	1.00 – 4.00
Possibility of risk to the professional (not at all likely/totally likely)	100.00 (9)	-	-	1.00 [1.00 ; 1.50]	1.00 – 4.00
Effectiveness of the intervention for the care of the person with type 2 diabetes (no effectiveness at all/totally effective)	-	11.11 (1)	88.89 (8)	10.00 [8.00 ; 10.00]	6.00 – 10.00
Understanding of the mechanism of action (totally incomprehensible/totally comprehensible)	-	11.11 (1)	88.89 (8)	9.00 [8.50 ; 10.00]	6.00 – 10.00
General acceptability (Completely unacceptable/completely acceptable)	-	11.11 (1)	88.89 (8)	9.00 [8.00 ; 10.00]	6.00 – 10.00
Feasibility					
Is time-consuming for application (almost no time/extremely long time)	11.11 (1)	33.33 (3)	55.56 (5)	8.00 [5.50 ; 9.50]	3.00 – 10.00
Requires ongoing support and supervision (almost no support and supervision/intense support and supervision)	11.11 (1)	-	88.89 (8)	10.00 [8.50 ; 10.00]	3.00 -10.00
Requires additional human resources (no additional human resources/substantial increase in human resources)	22.22 (2)	44.45 (4)	33.33 (3)	6.00 [3.00 ; 8.00]	1.00 – 9.00
Requires additional material resources (no additional material resources/substantial increase in material resources)	88.89 (8)	-	11.11 (1)	5.00 [3.50 ; 6.50]	1.00 – 9.00
Cost-effectiveness (not at all cost-effective/totally cost-effective)	-	22.22 (2)	77.78 (7)	10.00 [7.50 ; 10.00]	6.00 – 10.00

*IQR = Interquartile Range; §Min-Max= minimum-maximum values

## Discussion

This study contributed to obtaining stakeholders' feedback on the preliminary proposal for the Dia-D Program, with the purpose of validating a complex intervention based on theoretical and conceptual foundations, centered on the individual and family. The main results demonstrated acceptability and feasibility, with reservations that should be considered in depth due to the risk of compromising the proposal's sustainability. 

The sample of participants consisted of health professionals from three distinct categories—nurses, nutritionists, and pharmacists—in similar proportions. Almost all were women, around 40 years old, held specialist qualifications, and had less experience as diabetes specialists than in their overall professional careers. 

Regarding the 14 acceptability and feasibility criteria assessed, consensus was reached on the program being suitable for managing type 2 diabetes, comprehensible, acceptable, and effective, with no risks to individuals or health professionals. It requires minimal additional material resources, though it does require ongoing support and supervision. These findings indicate that, from the professionals' perspective, the proposal is conceptually robust, capable of being understood in terms of its assumptions and potential benefits for individuals with diabetes. The need for ongoing support and supervision may be related to the complexity and novelty of the activities involved in self-management education, such as the use of behavior change techniques. 

No consensus was reached on overall appreciation of the program, the effort and time required for its application, ease/difficulty of application, the demand for additional human resources, or its cost-effectiveness. This uncertainty regarding these assessed criteria is quite consistent with these professionals' work context, as implementing the proposal would imply changes in their operational dynamics. Changes within workplace environments have been widely studied for their destabilizing effects on interpersonal relationships and individual human responses among workers, with various ways proposed to facilitate them^34^. 

Furthermore, the Dia-D Program, with its focus on developing skills for type 2 diabetes self-management, represents a shift from the predominant paradigm of health education in Brazil, which is centered on the vertical transmission of knowledge and reactive responses to problems presented by individuals during healthcare. Therefore, implementing the program requires the involvement and commitment of the health professionals to expand their own competencies to promote diabetes self-management, which entails informed decision-making, self-care behaviors, problem-solving abilities, and active patient participation in their own care^4^. 

It is worth noting that, by incorporating training for health professionals as part of the proposal's implementation, the complexity of diabetes self-management education is also considered from the health professionals' perspective; they will need to be motivated to learn new educational approaches for person- and family-centered care, as well as practical application of behavior change techniques. This added value to professional competence has the potential to extend to the health service and underscores a real need for institutional appreciation and support for the proposal. 

Comparing the results obtained with those of other studies is hindered by the lack of research that evaluated the acceptability and feasibility of intervention proposals for diabetes self-management. In studies with this purpose, feasibility is measured through pilot testing in mixed-methods clinical trials, obtaining metrics such as recruitment, retention, participation, and completion rates; examples include the HEAL-D and EXTEND programs^35^-^36^. 

Facilitating the implementation of diabetes self-management education in clinical practice is a complex process that involves transformational leadership, where leaders value innovation, have a clear vision of the direction they intend to lead the organization, and guide team efforts accordingly^37^. Theoretical-methodological models that support proposals for improvement in healthcare practice have facilitated the change processes by providing well-defined operational steps and technical support^38^. 

Another important point is that diabetes self-management education is not a usual practice in various health services in Brazil. A study evaluating the structure of 49 Basic Health Units in the city of Pelotas (PR) for diabetes care identified that 46.9% had a self-care program and 44.9% offered continuous education to improve diabetes control. That evaluation aimed to identify how health management was organized to meet the Chronic Care Model, as recommended by public health policies for managing non-communicable chronic diseases^39^. 

Translating scientific knowledge into clinical practice remains a challenge in health services, even in developed countries. Reflections on this problem attribute barriers to diabetes self-management education access to low investments in education compared to medication treatments and technological resources, as well as to low valuation of the educational process by both people with diabetes and some doctors. Compounding this is the limited willingness of these professionals to share decision-making with the people they assist, as they view their social role as that of a specialist authority^37^,^40^. 

The competence of diabetes educators in promoting diabetes self-management needs to reflect the evolution of their professional role. Along with its evolution, this role has become complex and dynamic, shifting from one centered solely on information transmission to one focused on facilitating an active teaching-learning process, collaborating with the learner to achieve positive health outcomes^41^. To act in this manner, educators need to focus on developing their own knowledge, skills, and attitudes. As part of this effort, various organizations have developed desired competency profiles. 

In 2020, the Association of Diabetes Care & Education Specialists (ADCES) updated the term "diabetes educator" to "diabetes care and education specialist," defined as "a specialist who, as an integral member of the care team, provides collaborative, comprehensive, and person-centered care and education to people with diabetes and related conditions." Additionally, the ADCES defined a competency profile to be developed by professionals based on six domains: clinical management practice and integration, communication and advocacy, person-centered care and counseling across the lifespan, research and quality improvement, system-based practice, and professional practice^42^. 

Recently, the Federal Nursing Council of Brazil regulated the activities of nurses in diabetes care and education, defining general and specific duties and competencies for this role. Among these, the "knowledge of pedagogical strategies for diabetes education encompassing the seven self-care behaviors, psychosocial barriers, behavior change, adherence, and identification of support networks" stands out^43^. 

Analyzing the data obtained in this research and the set of recommendations from the specialized literature, it can be inferred that the professionals involved were aware of the importance and appropriateness of the Dia-D Program, recognizing the value of its theoretical and conceptual foundation and the positive effects it would bring to people with diabetes. However, they also recognized that the effective implementation of the Dia-D Program would require changes not yet achieved by the team, such as a larger contingent of professionals and managerial structures and processes that facilitate program sustainability. 

Both facilitating aspects and identified threats reflect these professionals' understanding of the impacts that paradigmatic shifts can achieve. Certainly, the results of this research should support the effective, efficient, and sustainable implementation of the Dia-D Program, as well as any other programs using the proposed design in the intervention development phase. It should be noted that proposals' implementation resources would be provided by the institution itself, which, as a private healthcare provider interested in fostering client loyalty among users and doctors would project a differentiated approach to caring for people with diabetes. 

Among the limitations of this study are those intrinsic to cross-sectional studies and the fact that the study sample did not include other stakeholders, such as managers and people with type 2 diabetes who are users of the health service. Additionally, the evaluation criteria were assessed only quantitatively, which may have prevented the identification of other barriers to the program's implementation from the participants' perspective. Despite the small number of participants, the sample consisted of all professionals from the institution involved in cring for people with diabetes. The results obtained are not generalizable, as they reflect the perspective of a single group of professionals within a specific health service. 

## Conclusions

The study showed that the Dia-D Program, although favorably regarded by health professionals on several acceptability criteria, has implementation barriers. Among these barriers were the effort, difficulty, and time required to implement the program, the need for additional human resources, and uncertainty regarding its cost-effectiveness. 

These suggest the need for a systematic implementation strategy for the proposal aimed at enhancing efficacy and sustainability. This study will contribute to the realization of a future clinical study involving health service users, designed to test the efficacy and effectiveness of the program. The data obtained can also support discussions with the institution's managers to overcome the identified barriers, to restructure work dynamics and staffing levels necessary for the program's implementation and sustainability. 
